# Supplementary material for: Contact with Nature in Social Deprivation during COVID-19: The Positive Impact on Anxiety
Source: Int J Environ Res Public Health. 2023 Jul 14;20(14):6361. doi: 10.3390/ijerph20146361 (PMC10379001; doi:10.3390/ijerph20146361)
Supplement: Supplementary file 1 [file ijerph-20-06361-s001.zip › ijerph-2452084-supplementary.pdf]

LUMSA UNIVERSITÀ
1939 8OTTANTESIMO 2019

Department of Human Studies

QUESTIONNAIRE

We are conducting research on the activities and experiences of individuals during the first period of lockdown due to the coronavirus emergency, specifically the March–May 2020 lockdown, which was particularly severe.

We kindly ask you to share your thoughts on the statements you will find below. We are interested in your personal opinion on the matter.

It is important to answer all the questions, carefully following the instructions and respecting the order in which they are presented.

Please fill out the questionnaire by yourself.

The questionnaire is anonymous and intended solely for scientific purposes. Your responses will be treated with utmost confidentiality (Law of 31 December 1996 No. 675, art. 10).

Thank you for your cooperation!

Instructions

Below you will find a list of statements. Please indicate how much you agree (or disagree) with each of them.

There are no right or wrong answers. <u>What we are interested in is your personal opinion.</u>

To respond, select one of the numbers found near each statement, according to the following rating scale.

Strongly Disagree	Disagree	Somewhat Disagree	Neither Agree nor Disagree	Somewhat Agree	Agree	Strongly Agree
0	1	2	3	4	5	6

Examples

Riding a bicycle is fun.	0	1	2	3	4	5	6

If you strongly agree with this statement, you should select number four.

Pizza is the most delicious food in our country.	0	1	2	3	4	5	6

If you completely disagree with this statement, you should select number zero.

Section 1

Please indicate your level of agreement or disagreement with the following statements, thinking about the <u>first period of lockdown</u>.

Strongly Disagree	Disagree	Somewhat Disagree	Neither Agree nor Disagree	Somewhat Agree	Agree	Strongly Agree
0	1	2	3	4	5	6

01	I have spent time with my regular group of friends.	0 1 2 3 4 5 6
02	I have visited my loved ones.	0 1 2 3 4 5 6
03	I have spent time with my classmates or colleagues.	0 1 2 3 4 5 6
04	I have socialized with my usual circle of friends.	0 1 2 3 4 5 6
05	I have stayed connected with my loved ones.	0 1 2 3 4 5 6
06	My interactions with other people have decreased compared to before the coronavirus emergency.	0 1 2 3 4 5 6
07	I have been unable to socialize with others as much as I would have liked.	0 1 2 3 4 5 6
08	I have visited public gardens.	0 1 2 3 4 5 6
09	I have connected with nature.	0 1 2 3 4 5 6
10	I have spent time among nature.	0 1 2 3 4 5 6
11	I have taken walks in green areas.	0 1 2 3 4 5 6
12	I have explored green areas outside the city.	0 1 2 3 4 5 6
13	My contact with nature has decreased compared to before the coronavirus emergency.	0 1 2 3 4 5 6
14	I have been unable to visit green areas as frequently as I would have liked.	0 1 2 3 4 5 6

Please indicate your level of agreement or disagreement with the following statements, thinking about *the green spaces that you visited during <u>the first lockdown period from March to May2020</u>.*

15	Spending time here gives me a break from my day-to-day routine.	0 1 2 3 4 5 6
16	It's as if the environment has no boundaries.	0 1 2 3 4 5 6
17	The setting is fascinating.	0 1 2 3 4 5 6
18	Being here suits my personality.	0 1 2 3 4 5 6
19	Coming here helps me to get relief from unwanted demands on my attention.	0 1 2 3 4 5 6
20	There is a clear order in the physical arrangement of the elements.	0 1 2 3 4 5 6
21	There is much to explore and discover here.	0 1 2 3 4 5 6
22	It is easy to do what I want.	0 1 2 3 4 5 6

Now, think about yourself during the first lockdown period from March to May 2020.

23	The way I felt was unpleasant.	0 1 2 3 4 5 6
24	My life has been fulfilling.	0 1 2 3 4 5 6
25	I have been satisfied with every aspect of my life.	0 1 2 3 4 5 6

26	I have not felt attractive.	0	1	2	3	4	5	6
27	I have been able to adapt to everything I wanted.	0	1	2	3	4	5	6
28	I have felt mentally fully awake.	0	1	2	3	4	5	6
29	I have found beauty in some things.	0	1	2	3	4	5	6
30	I have not had particularly positive thoughts.	0	1	2	3	4	5	6
31	I felt calm.	0	1	2	3	4	5	6
32	I felt secure.	0	1	2	3	4	5	6
33	I felt nervous.	0	1	2	3	4	5	6
34	I felt regretful.	0	1	2	3	4	5	6
35	I felt at ease.	0	1	2	3	4	5	6
36	I felt upset.	0	1	2	3	4	5	6
37	I have taken more medications for my psychological well-being compared to before the COVID-19 emergency.	0	1	2	3	4	5	6

Section 2

Now, please rate your level of agreement or disagreement with the following statements, considering yourself as a person in general. Please use the scale provided below.

Strongly Disagree	Disagree	Somewhat Disagree	Neither Agree nor Disagree	Somewhat Agree	Agree	Strongly Agree
0	1	2	3	4	5	6

38	I often feel a sense of oneness with the natural world around me.	0 1 2 3 4 5 6
39	I think of the natural world as a community to which I belong.	0 1 2 3 4 5 6
40	I recognize and appreciate the intelligence of other living organisms.	0 1 2 3 4 5 6
41	I often feel disconnected from nature.	0 1 2 3 4 5 6
42	When I think of my life, I imagine myself to be part of a larger cyclical process of living.	0 1 2 3 4 5 6
43	I often feel a kinship with animals and plants.	0 1 2 3 4 5 6
44	I feel as though I belong to the Earth as equally as it belongs to me.	0 1 2 3 4 5 6
45	I have a deep understanding of how my actions affect the natural world.	0 1 2 3 4 5 6
46	I often feel part of the web of life.	0 1 2 3 4 5 6
47	I feel that all inhabitants of Earth, both human and non-human, share a common ‘life force’.	0 1 2 3 4 5 6
48	Just as a tree can be part of a forest, I feel embedded within the broader natural world.	0 1 2 3 4 5 6
49	When I think of my place on Earth, I consider myself to be a top member of a hierarchy that exists in nature.	0 1 2 3 4 5 6
50	I often feel like I am only a small part of the natural world around me, and that I am no more important than the grass on the ground or the birds in the trees.	0 1 2 3 4 5 6
51	My personal welfare is independent of the welfare of the natural world.	0 1 2 3 4 5 6
52	I often feel a sense of oneness with the natural world around me.	0 1 2 3 4 5 6

Socio-demographic data

1) What is the sex that was assigned to you at birth?

☐ Female ☐ Male

2) What is your age?________________years old

3) What is your city of residence? ____

4) What is your educational qualification? *(Select only one response)*

☐ Elementary School Diploma - Lower Secondary School Diploma

☐ Upper Secondary School Diploma Superiore

☐ Bachelor's Degree

☐ Master's Degree or equivalent

☐ Doctorate / Master's Degree / Specialization School

5) What is your main occupation?

☐ Student ☐ Freelancer ☐ Manager

☐ Employee ☐ Teacher ☐ Craftsman/Craftswoman

☐ Trader ☐ Skilled Worker ☐ Unskilled worker

☐ Homemaker ☐ Enterpreneur

Other (Please Specify) ....……

Thank you for your cooperation!
